# Challenges in melanoma research and increased melanoma risk call for novel methodological and therapeutic/protective strategies: the case of procyanidin C1 targeting redox metabolism

**DOI:** 10.3389/fphar.2026.1819387

**Published:** 2026-04-29

**Authors:** Sinemyiz Atalay Ekiner, Agnieszka Gęgotek

**Affiliations:** Department of Analytical Chemistry, Medical University of Bialystok, Mickiewicza, Białystok, Poland

**Keywords:** climate change, melanoma, natural compounds, procyanidin C1, skin, UV exposure

## Introduction

Melanoma accounts for a small percentage of skin cancer cases but it is the most aggressive type, with the highest mortality rate. Moreover, due to the complex and highly heterogeneous biology of melanoma, therapeutic options remain limited (Ng et al., 2022; Naimy et al., 2024; Atalay Ekiner et al., 2025). Furthermore, literature data indicate that climate change increases the risk of developing melanoma (Watson et al., 2024). The challenges arising from the complex melanoma biology and its pharmacotherapy urgently call for the development of new research strategies, including optimized experimental systems as well as novel protective/therapeutic approaches (Matias et al., 2021). Here, we highlight the largely unknown interplay between rising melanoma risk and climate change, as well as the need for improved research strategies and the potential of natural compounds in novel prevention and therapeutic approaches. Natural compounds with redox-regulating activity, recently reviewed, show promise for counteracting skin cancers, including melanoma (Atalay Ekiner et al., 2025). While other compounds retain their potential, recent data particularly highlight procyanidin C1 (PCC1), as it shows promise for skin protection against melanomagenesis.

## Challenges in melanoma research

The pluripotent nature of the neural crest lineage makes neural crest stem cell-derived tissues particularly susceptible to a wide spectrum of tumors driven by diverse mutations, including melanoma (Khan et al., 2024). Melanoma etiology, presenting highly complex interplay between genetic and environmental factors, contributes to its aggressive metastatic character and resistance to conventional therapies. Understanding the complex molecular patterns underlying melanoma biological complexity influencing disease progression and therapeutic resistance is both challenging and critical for research aimed at developing effective therapeutic strategies, particularly those that consider individual variability.

Extensive research highlights the pivotal role of UV radiation and its oxidative effects as major contributors to melanocytic transformation and melanoma development (Gieniusz et al., 2024). In melanoma cells, oxidative stress acts paradoxically: it can drive tumor initiation but also limit vertical growth and metastasis. The attenuation of oxidative stress within the tumor microenvironment seems to constitute a key adaptive mechanism (Becker and Indra, 2023). Accordingly, the compounds with redox-regulatory properties offer considerable potential for developing protective/therapeutic strategies in melanoma. Together with that, considering the high heterogeneity observed in melanoma, it is particularly critical that all analyses of the biological effects of any compound – whether used alone or in combination therapies – especially at the *in vitro* preclinical level (with a focus on future studies investigating macromolecules of functional importance for cellular metabolism and associated signaling pathways, including the proteome, lipidome, and metabolome studies) – be conducted in detail across multiple melanoma cell lines, allowing for comparative analyses. Moreover, it is important to note that, in addition to the distinct melanoma cells, neighboring cells should be included as part of this complex analysis. Here, fibroblasts, in particular, have been extensively reported in the literature to play a key role in supporting melanoma progression and metastasis (Wądzyńska et al., 2023). Additionally, keratinocytes emerge as skin cells that play critical roles in the primary microenvironment responsible for melanoma spread, due to their position surrounding epidermal melanocytes attached to the basement membrane (Marrapodi and Bellei, 2024).

Previously, a study showed that tumor-derived fibroblasts from metastatic melanomas strongly impair NK cell function, by reducing NK cell-mediated killing of melanoma cells through prostaglandin E2 signaling, either constitutively or upon NK cell-induced stimulation (Balsamo et al., 2009). In contrast, normal skin fibroblasts only partially affect NK cell phenotype and function. A recent study analyzing the transcriptional signatures of cancer-associated fibroblasts (CAFs) and normal fibroblasts identified differences between primary tumor- and metastasis-specific CAFs. The findings indicate tumor-dependent signaling between neutrophils and CAFs, mediated via SAA3-and IL1β-related pathways, which may regulate neutrophil recruitment and t-NET formation (Tauch et al., 2025). Moreover, phenotypic alterations in keratinocytes following exposure to the melanoma-derived secretome have been shown to contribute significantly to early neoplastic transformation by modulating tumor responses and shaping a microenvironment that supports melanoma progression (Marrapodi et al., 2025). Although no direct comparative example is currently available, these findings suggest that particularly early-stage (2D) single type-cell studies – despite their simplicity and associated advantages for investigating molecular/biochemical mechanisms – may not adequately capture the complexity of melanoma biology and may create gaps or misinterpretations in subsequent *in vivo* and clinical studies. Here, from a therapeutic perspective, the role of any candidate compound in modulating cellular responses within such a complex signaling microenvironment becomes especially critical, particularly when compared to responses observed in the absence of this microenvironment. This highlight the need to assess potential compounds not only in melanoma cells but also in neighboring cells, particularly using advanced models such as 3D skin models (with co-culturing) to improve experimental validation and accuracy. This is also crucial for early diagnostic studies targeting transcriptomic, proteomic, and lipidomic differences. Moreover, further methodological advancements in *in vivo* functional screening and multi-omics analyses are necessary – for example, studies identifying α-2,3-sialylation as a critical factor for melanoma maintenance (Agrawal et al., 2025) – taking into account melanoma heterogeneity, as well as the responses of neighboring cells alongside melanoma cells.

## Melanoma risk under climate change

It’s well-known that UVR – promoting DNA damages and specific mutations which are particularly associated with melanoma as well as triggering reactive oxygen and nitrogen species generation and associated downstream oxidative damage to macromolecules – can disrupt cellular homeostasis and metabolism, and even these metabolic changes ultimately may lead to the development of skin cancers (Pfeifer, 2020; Sun et al., 2020; Tang et al., 2024; Wei et al., 2024). Growing evidence indicates that climate change may increase the risk of both melanoma, arising from melanocyte dysfunction, and non-melanoma skin cancers derived from epidermal cell abnormalities (Khan et al., 2022), through UVR exposure, higher temperatures, and worsening air pollution (Watson et al., 2024). Within this framework, the association between UV exposure and melanoma development is of particular importance. This is due to evidence showing that climate change affects UV irradiance patterns and increases cumulative annual UV exposure (Baldermann et al., 2023). Although there is still a significant gap requiring multidisciplinary and systematic investigation of the relationship between climate change, UVR exposure, and skin cancer development, the issue is a well-founded and potentially serious concern. Alongside the need to develop and implement effective climate change solutions, it is also essential to consider this issue in relation to its observed social-economic impacts and the systemic barriers to global health (Goniewicz et al., 2025).

## The potential of natural compound-based therapeutic and protective applications

While non-melanoma skin cancers are the most prevalent, melanoma remains the most aggressive and lethal form of skin cancer (Sharma et al., 2024). The biological complexity of melanoma, together with the limited efficacy of conventional melanoma therapies, necessitates the development of novel or adjunctive treatment strategies, among which natural compounds have emerged as promising candidates. Numerous research indicates that natural compounds and their structural analogues, including polyphenols, vitamins, alkaloids, and cannabinoids, exhibit anti-cancer effects and may play a role in preventing and treating skin cancer. Although these compounds possess favorable biochemical properties, including the ability to modulate redox signaling, inflammation, and cell survival in target cells (Fernandes et al., 2023), as well as advantages in large-scale production and lower toxicity compared with synthetic compounds, it is also important to acknowledge their existing limitations. Technical challenges related to screening, isolation, characterization, and optimization remain (Atanasov et al., 2021), highlighting the need for methodological improvements. Additionally, challenges in standardizing extracts from natural sources – such as lipid extracts from plant/marine sources – require detailed quantitative analyses of active ingredients during preclinical evaluation, while also considering resource collection timing and location, production conditions, and environmental sustainability.

Despite these limitations and methodological challenges, particularly the redox-regulating properties of natural compounds, coupled with their reduced side effects relative to synthetic alternatives, underscore their high potential in melanoma and other oxidative stress-related diseases. Given the aggressive nature of melanoma, this potential – especially in terms of protection against melanomagenesis compared to therapeutic efficacy – is evident and merits further in-depth investigation. Particular attention should be given to investigating the effects of antioxidant compounds in the skin on tumor initiation, through cell differentiation-related signaling pathways as well as the changes within the tumor microenvironment, while considering genomic variability. Furthermore, concerning therapeutic effectiveness and given the limitations observed in the clinical effectiveness of therapies targeting oxidative stress (Davies and Holt, 2018; Forman and Zhang, 2021; Chen et al., 2025), their use in combination with approaches such as immunotherapy and nanomedicine (Liang X. et al., 2025) is presenting greater benefits than relying on them as standalone treatments.

Moreover, recent studies support this potential in comparison to synthetic and natural compounds. Although synthetic compounds predominate in early-phase clinical trials, their relative representation declines by phase III. Conversely, the proportion of natural products and hybrids increases from phase I to phase III (Domingo-Fernández et al., 2024). Thus, natural compounds demonstrate clear therapeutic/protective potential and offer promising prospects for future clinical use. Procyanidin C1 (PCC1) is one such compound, exhibiting protective and therapeutic potential against melanoma.

## PCC1: a candidate for therapeutic and protective intervention in melanoma

We recently reviewed promising natural compounds for the prevention and treatment of skin cancer in general, including melanoma (Atalay Ekiner et al., 2025). Based on the latest data, PCC1 emerges as a particularly promising compound due to its significant biological activities observed so far. In particular, current findings provide a more comprehensive view of PCC1 as a single compound, highlighting its efficacy in skin-related indications (including both cancerous and healthy skin cells), as well as in non-cutaneous cancers including colon, breast, lung and gastric cancers.

PCC1 is a naturally occurring B-type proanthocyanidin flavonoid found in grapes. Its phenolic structure suggests anti-inflammatory, redox-regulating, and microbiome-modulating effects (Xu et al., 2021; Wang et al., 2024). Firstly, it has been demonstrated that it exerts a direct regulatory effect on the redox status of UVB-exposed HaCaT cells by reducing reactive oxygen species and lipid peroxides generation as well as modulating the metalloproteinase-1 (MMP1) and wingless-related integration site (Wnt) signaling pathways (Liu et al., 2024). This is primarily due to PCC1’s protective effect on skin keratinocytes against UVB-induced oxidative damage, which may play a key role in preventing melanomagenesis. It is worth noting that, although both UVA and UVB play a role in melanoma formation, UVB markedly accelerates melanoma onset through the generation of dimeric DNA photoproducts, predominantly cyclobutane pyrimidine dimers (Jin et al., 2022). Therefore, this activity, particularly against UVB, suggests considerable potential, given the critical role of keratinocytes in establishing the initial microenvironment that contributes to melanoma development and early spread, as mentioned above. Nevertheless, further studies are required to investigate its effects in greater detail, particularly at the molecular level, including DNA, protein, and lipid oxidative modifications as well as specific DNA mutations.

Additionally, PCC1-mediated modulation of MMP1 and Wnt signaling in keratinocytes highlights the compound’s ability to interfere with pathways involved in melanoma development by altering molecular signaling in keratinocytes surrounding melanocytes. It’s known that Wnt signaling, involving stem cell maintenance, plays a critical role not only in tumorigenesis, but also cancer progression, and therapeutic resistance in melanoma growth and metastasis (Liang J. et al., 2025). On the other hand, UVB-exposed keratinocytes express several MMPs that promote inflammatory responses, degrade extracellular matrix components, and facilitate cell mobilization, thereby contributing to remodeling of the epidermal microenvironment – processes that play important roles in melanoma initiation and progression (Marrapodi and Bellei, 2024).

Furthermore, PCC1 ability to modulate the microbiome and associated host metabolic response also particularly stands out (Wang et al., 2024; Liu et al., 2026). Importantly, recent studies highlight the microbiome as a key factor shaping (both local and systemic) immune landscapes, influencing tumor development and modulating therapeutic responsiveness in melanoma (Bautista et al., 2025). This perspective offers important insights for precision immuno-oncology, especially considering the complex biology of melanoma and the ongoing challenges associated with its treatment. Thus, although the potential of other natural compounds remains relevant (Atalay Ekiner et al., 2025), and considering the increasing risks in melanoma incidence, along with the rapidly changing environmental conditions, ongoing challenges in both melanoma research and treatment, we highlight PCC1 here due to the significant features identified in recent studies.

**FIGURE 1:**
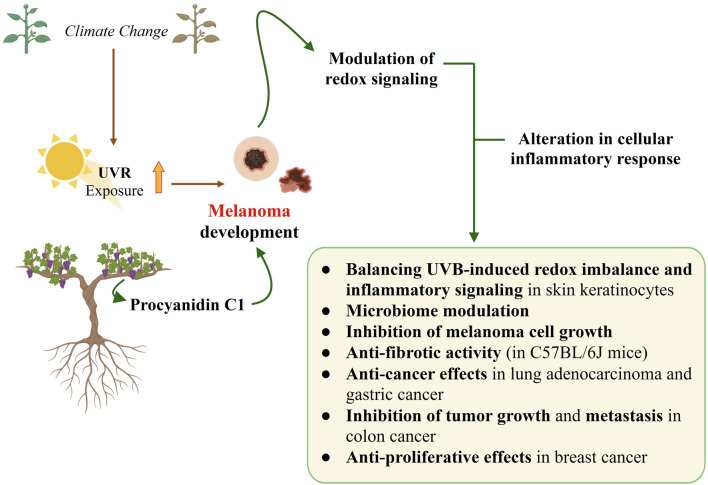
Climate change can increase the risk of developing melanoma by increasing skin exposure to ultraviolet radiation (UVR). Procyanidin C1, a natural compound found in grapes, is a promising compound to combat melanoma by modulating intracellular redox signaling and the associated pathways involved in inflammation, cell survival, and differentiation. Despite the limited number of studies, those investigating the activity of PCC1 in the context of melanoma as well as other cancer types, the available findings warrant further detailed analysis. The figure was created in BioRender.

Moreover, its ability to accumulate at biological membrane interfaces and self-assemble into higher-order oligomers indicates the possibility of new implications for future therapeutic strategies that may arise from membranotropic mechanisms (Villalaín, 2022). This characteristic is especially important for permeation across the skin lipid barrier, offering promising opportunities for future therapeutic development. Moreover, what is very important is the fact that phenolic compounds, including PCC1, possess redox-active properties that enable them to directly modulate cellular redox homeostasis and associated signaling pathways involved in inflammation, cell survival, and differentiation (Kim et al., 2023). This makes them promising candidates for combating oxidative stress-related diseases, with melanoma representing one of the most critical examples (Figure 1).

PCC1 has been shown to reduce ROS generation and alleviate oxidative stress at concentrations of 12.5–50 μmol/L in pancreatic β-cells exposed to H_2_O_2_ (Sun et al., 2016). Conversely, PCC1 has been reported to increase ROS levels, induce mitochondrial dysfunction, and modulate apoptosis at a concentration of 100 μM in senescent PSC27 mouse cells (Xu et al., 2021). These findings suggest a dual role for PCC1, acting as both an antioxidant and a pro-oxidant, depending on the concentration and cell type. As a phenolic compound, its pronounced redox-modulating activity is of particular significance and holds potential for novel therapeutic applications. This is especially relevant in skin cancers, particularly in melanoma, where ROS and inflammation signaling, which interact with each other (Morris et al., 2022), act as key contributors (Xian et al., 2019). Thus, agents with antioxidant properties may have the greatest utility in chemoprevention whereas those with pro-oxidant properties may be better suited for treatment (Becker and Indra, 2023). The purpose of this review is to provide an overview of oxidative stress in melanoma, and how the antioxidant system may be manipulated in a therapeutic context for improved efficacy and survival. However, evaluating the effects of the compound – here, PCC1 – on other skin cell types beyond melanoma cells is important in this context, particularly regarding redox-related inflammation and cellular differentiation and survival.

Despite the limited number of studies, those investigating the activity of PCC1 in the context of melanoma as well as other cancer types, the available findings warrant further detailed analysis. Additionally, anti-fibrotic activity of PCC1 has been shown in in male C57BL/6J mice (Wang et al., 2025). As fibrosis has been acknowledged as a key contributor to the development of multiple cancers, including melanoma playing a major role in drug adaptation and therapeutic resistance (Diazzi et al., 2020), any potential regulatory role of this compound in melanoma warrants attention. Moreover, two studies have shown that PCC1 treatment suppresses melanoma growth (Bae et al., 2020) and counteracts the age-dependent reduction in the tumor-killing capacity of CD8^+^ T cells (Duan et al., 2024). Furthermore, the efficacy of PCC1 has also been demonstrated in other types of cancer, highlighting its promising therapeutic potential, including the inhibition of tumor growth and metastasis in colon cancer (Lv et al., 2024) and antiproliferative activity in breast cancer (Koteswari et al., 2020).

## Conclusion and closing remark

While evidence on PCC1, especially in melanoma, is still limited, the available findings, combined with its redox-regulatory properties, indicate that PCC1 may be a promising candidate for melanoma prevention and therapeutic applications. Further studies need to investigate the compound’s mechanism of action and evaluate its effects across different melanoma subtypes. In addition, the compound’s effects within the multicellular skin environment and its complex molecular signaling should be investigated, including its impact not only on melanoma cells but also on other skin cell types. In particular, beyond different melanoma cell types, the effects on skin keratinocytes and fibroblasts should be systematically analyzed (related to both melanoma development and metastasis and progression), especially with respect to cellular antioxidant, inflammatory, and death/differentiation response, in combination with omics-based approaches. Such analyses should be conducted using 3D *in vitro* co-culture systems, as well as *in vivo* skin models. Its potential toxicity in healthy human cells and its ability to elicit a pro-inflammatory response in the bloodstream should also be carefully evaluated.

The existing gap in the literature regarding the effects of this compound on lipid metabolism also warrants attention. In this context, it is important to investigate how the compound modulates lipid metabolism (including fatty acids, phospholipids, ceramides, and sphingolipids), along with the associated signal transduction pathways. Such analyses, particularly in the context of cancer-related lipid metabolic reprogramming, may provide critical insights into the extent to which PCC1-mediated processes contribute to lipid metabolism-associated cell death and to alterations in tumor cell proliferation, invasion, and metastasis, thereby supporting the development of novel strategies.

Finally, beyond their pharmacotherapeutic potential, factors such as the accessibility, characterization, scalable production, and sustainable use of natural compounds, including PCC1 as given here and others, are critical considerations. These aspects, combined with future multidirectional analyses and the development of strategies to mitigate the health impacts of climate change – particularly its harmful consequences in the context of melanoma specifically, as well as other cancers and infectious diseases – are essential for advancing research and public health outcomes. Accordingly, integrative and interdisciplinary research efforts will be crucial to translate these insights into practice. Ultimately, such efforts may enable the identification of novel biomarkers and targets, supporting more precise risk assessment and the development of targeted prevention and treatment approaches.
